# Predictive modeling of aspirin‐triggered resolvin D1 pharmacokinetics for the study of Sjögren's syndrome

**DOI:** 10.1002/cre2.260

**Published:** 2019-12-19

**Authors:** Venkata Kashyap Yellepeddi, Olga J. Baker

**Affiliations:** ^1^ Division of Clinical Pharmacology, Department of Pediatrics, School of Medicine University of Utah Salt Lake City Utah; ^2^ Department of Pharmaceutics and Pharmaceutical Chemistry, College of Pharmacy University of Utah Salt Lake City Utah; ^3^ School of Dentistry University of Utah Salt Lake City Utah

**Keywords:** computer modeling, hyposalivation, inflammation, pharmacokinetics, physiologically based pharmacokinetic (PBPK) modeling and Sjögren's syndrome, resolution

## Abstract

**Objectives:**

Sjögren's syndrome (SS) is an autoimmune disease that causes chronic inflammation of the salivary glands leading to secretory dysfunction. Previous studies demonstrated that aspirin‐triggered resolvin D1 (AT‐RvD1) reduces inflammation and restores tissue integrity in salivary glands. Specifically, progression of SS‐like features in NOD/ShiLtJ mice can be systemically halted using AT‐RvD1 prior or after disease onset to downregulate proinflammatory cytokines, upregulate anti‐inflammatory molecules, and restore saliva production. Therefore, the goal of this paper was to create a physiologically based pharmacokinetic (PBPK) model to offer a reasonable starting point for required total AT‐RvD1 dosage to be administered in future mice and humans thereby eliminating the need for excessive use of animals and humans in preclinical and clinical trials, respectively. Likewise, PBPK modeling was employed to increase the range of testable scenarios for elucidating the mechanisms under consideration.

**Materials and methods:**

Pharmacokinetics following intravenous administration of a 0.1 mg/kg dose of AT‐RvD1 in NOD/ShiLtJ were predicted in both plasma and saliva using PBPK modeling with PK‐Sim® and MoBi® Version 7.4 software.

**Results:**

The model provides high‐value pathways for future validation via in vivo studies in NOD/ShiLtJ to corroborate the findings themselves while also establishing this method as a means to better target drug development and clinical study design.

**Conclusions:**

Clinical and basic research would benefit from knowledge of the potential offered by computer modeling. Specifically, short‐term utility of these pharmacokinetic modeling findings involves improved targeting of in vivo studies as well as longer term prospects for drug development and/or better designs for clinical trials.

## 1 INTRODUCTION

Sjögren's syndrome (SS) is an autoimmune disease that causes chronic inflammation of the salivary glands leading to secretory dysfunction. It is considered to be the second most common rheumatic autoimmune disorder after rheumatoid arthritis (Fox, 2005), with prevalence ranging from 0.03% to 2.7% depending on geographical region and the classification criteria used (Patel & Shahane, 2014). The causes or cure for SS are unknown; however, the disease is characterized by uncontrolled inflammation of the salivary glands, suggesting alteration in the resolution of inflammation in this organ (Baker, 2016; Baker et al., 2008; Bhattarai, Junjappa, Handigund, Kim, & Chae, 2018; Bombardieri et al., 2012; C. S. Wang & Baker, 2018; C. S. Wang, Wee, Yang, Melvin, & Baker, 2016).

Resolution of inflammation is an actively regulated process mediated in part by a family of lipid‐based specialized proresolving mediators (SPM) that include resolvins, maresins, lipoxins, protectins, and their aspirin‐triggered (AT) forms that are comparable in their properties with naturally occurring SPM (reviewed in Chiang & Serhan, 2017; Recchiuti et al., 2014; Serhan, 2008, 2014, 2017a, 2017b; Serhan & Chiang, 2008) but have a longer half‐life as described below (Sun et al., 2007). Studies of SPM and AT forms within the salivary glands have been largely confined to a single resolvin (AT‐RvD1) that has shown particular promise for treating hyposalivation.

The current studies were conducted to extend our earlier referenced findings regarding the utility of AT‐RvD1 (aspirin‐triggered 17R epimer of resolvin D1) for hyposalivation (C. S. Wang, Maruyama, Easley, Trump, & Baker, 2017) treatment with a particular emphasis on describing its plasma pharmacokinetics (PK) in mice and humans. To that end, physiologically based pharmacokinetic (PBPK) computer modeling was employed to increase the range of testable scenarios for elucidating the mechanisms under consideration with the aim of conducting future in vivo studies to validate the predictive validity of the computer models employed herein, for which details are provided below.

Understanding the PK of candidate drugs has become an essential part of the drug development process (Walker, 2004), as it is estimated that approximately 10% to 40% of drug candidates fail due to a lack of optimal PK (Prentis, Lis, & Walker, 1988). The knowledge of PK provides vital information about the relationship between PK and pharmacodynamics (PD) of the candidate drugs, and such information can help in decision making about drug development progression, dose selection, and ultimately clinical study strategies (Sager, Yu, Ragueneau‐Majlessi, & Isoherranen, 2015). Traditionally, PK studies are performed in animals in preclinical stage and in humans during early stages (Phase I) of clinical trials, such experiments are time consuming and expensive, thereby adding significantly to the overall cost of drug development. However, PK costs have been streamlined in the past decade with the help of quantitative modeling and simulation, thereby allowing this approach to emerge as a powerful tool for prediction of PK at both preclinical and clinical stages of drug development (Chien, Friedrich, Heathman, de Alwis, & Sinha, 2005; Jiang et al., 2011; Sheiner & Steimer, 2000; Y. C. Wang et al., 2018).

**Figure 1:**
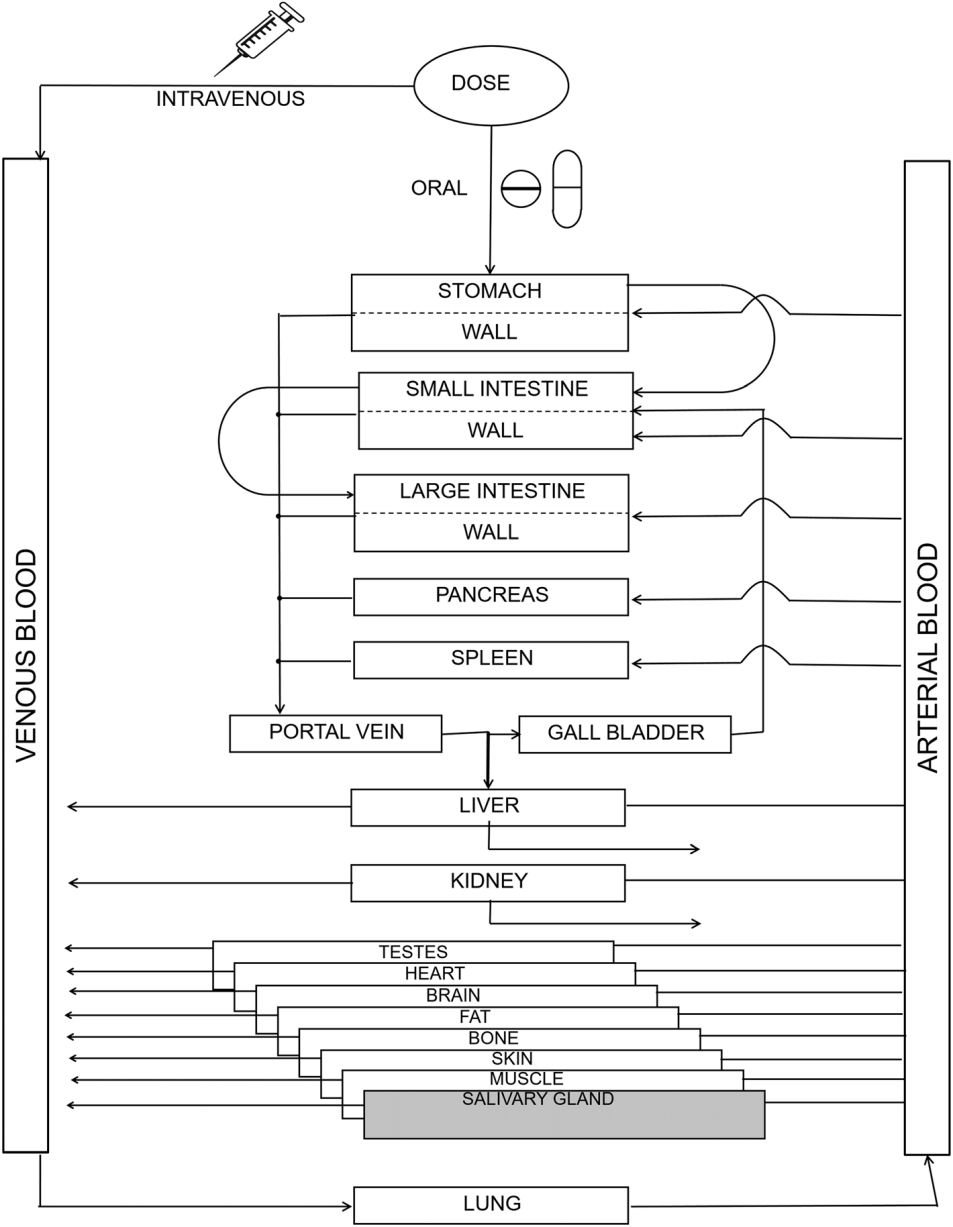
Structure of a physiologically based pharmacokinetic model: The organism is divided into a number of compartments, each representing a single organ. To describe the distribution of compounds in the body, the organs are connected via their arteries and veins to the arterial and venous blood pool. Intercompartmental mass transport occurs via organ‐specific blood flow rates. The organs are mathematically connected

**Figure 2:**
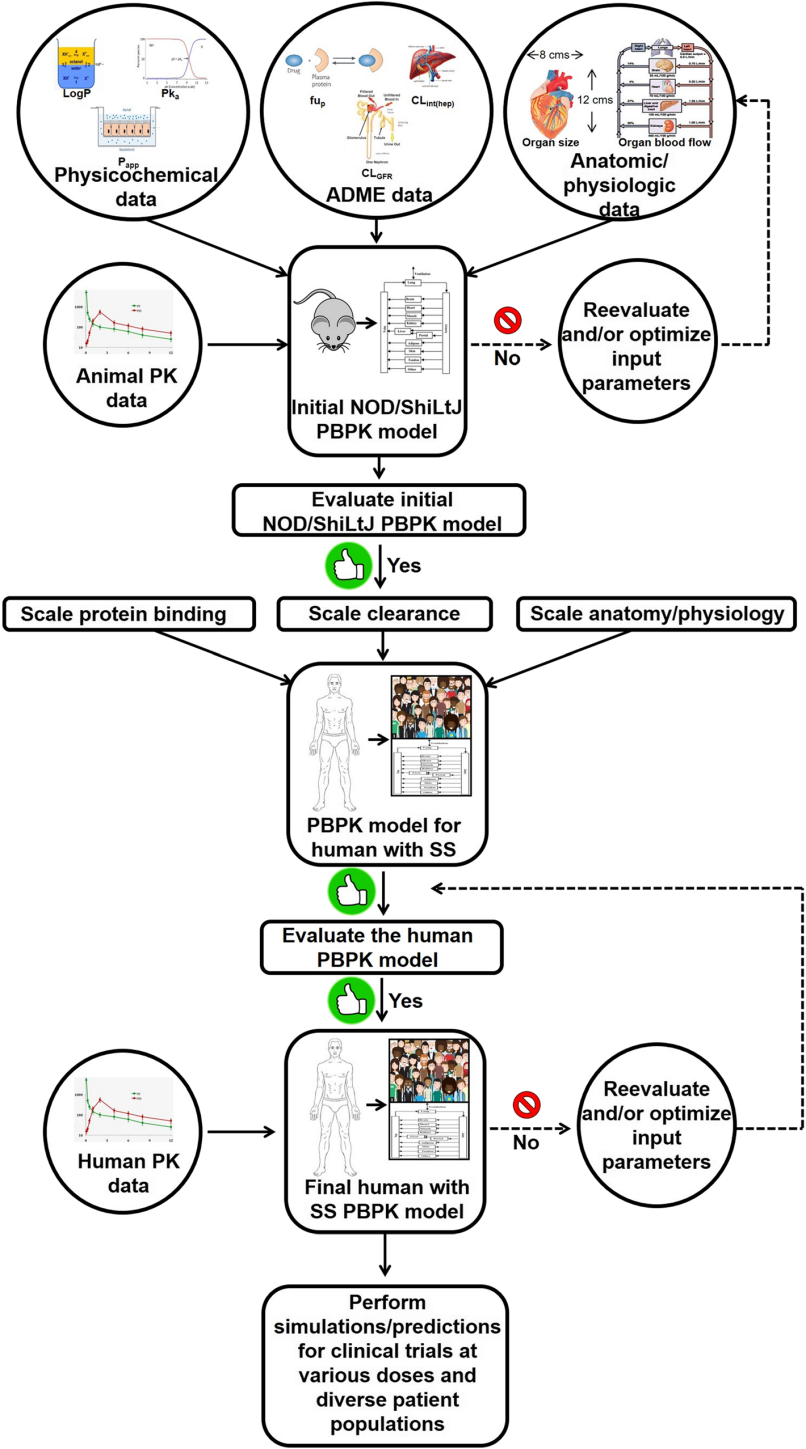
Workflow of PBPK model development for AT‐RvD1. The PBPK model for human with SS refers to the virtual population of the subjects whose anatomical physiological parameters reflect that of someone with SS. If the model‐predicted data does not agree with the observed experimental data, a parameter identification and optimization will be performed using the default tools provided by the PK‐Sim® software to identify the parameter responsible for the lack of accurate predicts and then its value will be optimized using the observed data until a goodness of fit is achieved between predicted versus observed data. AT‐RvD1, aspirin‐triggered resolvin D1; PBPK, physiologically based pharmacokinetic; PK, pharmacokinetics; SS, Sjögren's syndrome

PBPK is a contemporary modeling and simulation approach used in drug discovery to influence the selection of molecules based on their characteristics. PBPK modeling utilizes physiological and chemical‐specific parameters as well as mathematic equations to quantitatively describe in vivo disposition of drugs (Jones & Rowland‐Yeo, 2013; Yellepeddi et al., 2018). Moreover, they provide simulated concentration versus time profiles of a drug and its metabolite(s) in plasma or an organ of interest and simultaneously allow for estimation of maximum plasma concentrations, absorption kinetics, distribution kinetics, and drug elimination (Sager et al., 2015; Yellepeddi et al., 2018). The structure of the PBPK model used in this project with additional salivary gland compartment is provided in Figure 1. The simulated concentration–time profiles can aid in the selection of optimal sampling times or dosing strategies in various populations, including vulnerable subjects (Rowland, Peck, & Tucker, 2011). Additionally, the simulated concentrations can be linked to PD endpoints to allow for PK/PD simulations. Due to these advantages, there has been a rapidly growing interest in PBPK modeling in recent years in the pharmaceutical industry, academia, and regulatory agencies such as United States Food and Drug Administration and European Medicines Agency (Rostami‐Hodjegan, 2012; Rostami‐Hodjegan, Tamai, & Pang, 2012; Yellepeddi et al., 2018). The workflow of development of a PBPK model with its application for predicting pharmacokinetics of therapeutics for SS is provided in Figure 2.

## 2 MATERIALS AND METHODS

### 2.1 Model parameters

The drug‐specific information for the model parameters was obtained from both literature and using quantitative structure–property relationship calculations. The quantitative structure–property relationship calculations were performed using Chemicalize® by ChemAxon® Company, Cambridge, MA, USA. The physicochemical properties of AT‐RvD1 include molecular weight, LogP, pKa, the presence of halogens, and solubility at physiological pH 7.4. Other drug‐specific parameters that were collected for AT‐RvD1 are fraction unbound in plasma (mice and human), plasma protein to which AT‐RvD1 binds, and enzymes responsible for the metabolism of AT‐RvD1. The specific values of AT‐RvD1 used in building the model are provided in Table 1. The mouse‐specific model parameters for the NOD/ShiLtJ mice were obtained from Mouse Strain Datasheet‐001976 provided by the supplier, the Jackson Laboratory, Bar Harbor, ME, USA (Laboratory, 2018b). The NOD/ShiLtJ strain with SS‐like symptoms is a polygenic model used to evaluate the effectiveness of AT‐RvD1 for reduction of hyposalivation (Easley et al., 2015; C. S. Wang et al., 2017).

**Table 1:** Drug‐specific parameters of aspirin‐triggered resolvin D1 used for building the physiologically based pharmacokinetic model

Drug‐specific parameter	Value	Reference
Molecular weight	376.493 g/mol	(NCBI, 2019)
Formula	C_22_H_32_O_5_	(NCBI, 2019)
Composition	C (70.19%), H (8.57%), O (21.25%)	(NCBI, 2019)
Topological polar surface area	97.99 A°^2^	(NCBI, 2019)
pKa	4.47	QSPR^a^
Isoelectric point	1.44	QSPR
LogP	3.22	QSPR
Solubility at pH 7.4	3.1 mg/ml	QSPR

a QSPR—quantitative structure property relationships were calculated using Chemicalize® by ChemAxon®.

### 2.2 PBPK model building

We developed a whole‐body PBPK model for the AT‐RvD1 in NOD/ShiLtJ mice using the software PK‐Sim®, Version 7.4.‐Build 127 (Open Systems Pharmacology Suite, http://www.open-systems-pharmacology.com). The salivary gland and saliva compartments for NOD/ShiLtJ was added using the MoBi®, Version 7.4. The PBPK model was structured with 15 organs using mass balance differential equations describing drug entering and exiting the various organ compartment, and the link between physiologic spaces was blood circulation. NOD/ShiLtJ mouse body weight information was incorporated from the information obtained from the Jackson Laboratory (Laboratory, 2018a). The initial base model was built using the NOD/ShiLtJ mouse model, as we have PD evidence indicating that AT‐RvD1 reduces hyposalivation when administered intravenously. The endothelial surface area (SA) is needed for calculation of the rate of permeation through the endothelial barrier between plasma and interstitial space. The capillary endothelial SA is calculated by the organ vascularization method using the equation (Community, 2017; Niederalt et al., 2018; Willmann, Schmitt, Keldenich, Lippert, & Dressman, 2004)

$$\mathrm{SA}=k\cdot f_{\mathrm{vas},\;\text{organ}}\cdot V_{\text{organ}},$$

where *k* is the proportionality constant, *f*_vas, organ_ is the fraction of vascular space of an organ, and *V*_organ_ is the organ volume.

For distribution model, the partition coefficients for the organs were calculated using the PK‐Sim® standard model using the equation (Mavroudis, Hermes, Teutonico, Preuss, & Schneckener, 2018; pharmacology, 2017; Willmann et al., 2004)

$$K_{\text{organ}}=\left(F_{\text{water}}^{\text{organ}}+K_{\text{lipid}}F_{\text{lipid}}^{\text{organ}}+K_{\text{protein}}F_{\text{protein}}^{\text{organ}}\right)\cdot f_{u}^{\text{plasma}},$$

where $F_{x}^{\text{organ}}$ = volume fraction of water, lipid, and protein, *K*_*lipid*_ = lipid/water partition coefficient, *K*_*protein*_ = protein/water partition coefficient, and $f_{u}^{\text{plasma}}$ = free fraction in plasma. The partition coefficients *K*_*lipid*_ and *K*_*protein*_ are derived from input data of physicochemical properties of AT‐RvD1.

The pathway of AT‐RvD1 metabolism in vivo is a crucial component of the current model. It is reported that AT‐RvD1 is metabolized in vivo by the enzyme eicosanoid oxidoreductase (EOR), also known as 15‐prostaglandin dehydrogenase (Sun et al., 2007). Therefore, we incorporated abundance information of EOR in our initial mice PBPK model. The genetic information about EOR was obtained from the National Center for Biotechnology Information (NCBI), gene resources (NCBI, 2018). Furthermore, the kinetic parameters of enzymatic degradation *K*_m_ value of human EOR enzyme were obtained from the literature and incorporated in the model (Zhou, Yan, & Tai, 2001).

The NOD/ShiLtJ mouse PBPK model was extrapolated to humans after first taking into account the interspecies differences in physiological and chemical‐specific parameters. Specifically, for the latter, the model assumes a 30‐year‐old Caucasian American population with a mean body weight of 80.35 kg and height of 178.49 cm, consistent with population‐level data from the National Health and Nutrition Examination Survey dataset (NHANES, 1997).

### 2.3 Simulations in mice and humans

For performing population simulations of AT‐RvD1 pharmacokinetics in mice, a NOD/ShiLtJ population of 100 animals was created from an individual mouse. The weight for the population was set to vary from 12 to 30 g based on the data obtained from Mouse Strain Datasheet for NOD/ShiLtJ from the Jackson Laboratory (Laboratory, 2018a, 2018b). The concentrations of AT‐RvD1 in saliva and plasma as well as highly perfused organs such as the brain, heart, lung, liver, kidneys, and spleen were simulated within the mouse population studied. For simulations with the human population, 100 subjects with an equal proportion of sex with age ranging from 20 to 80 years were used, and anatomical and physiological parameters were adjusted by PK‐Sim® based on age, with studies conducted in the same fluids and organs noted above for mice (i.e., saliva, plasma, and a range of perfused tissues).

The predicted plasma concentration versus time curves of AT‐RvD1 after single intravenous bolus administration at a 0.1 mg/kg dose in NOD/ShiLtJ mice and humans were generated. The simulations were performed from time 0 to 24 hr.

### 2.4 Pharmacokinetic parameters

The PK parameters area under the curve (AUC), maximum concentration (*C*_max_), time at maximum concentration (*T*_max_), half‐life (*t*_1/2_), mean residence time, apparent volume of distribution (*V*_d_), and total plasma clearance were calculated using simulated plasma concentration and time curves that were generated between 0‐ and 24‐hr time points.

### 2.5 Sensitivity analysis

A sensitivity analysis for the parameters LogP, fraction unbound, pKa, plasma clearance, hematocrit, and volume of adipose tissue among others was performed with the sensitivity analysis tool provided with the PK‐Sim® software. Sensitivity analysis assesses parameters that could influence model performance. All input parameter values were replicated with a 10‐fold change in the mean to determine if any parameter significantly affected the output PK parameters AUC_0‐inf_ and total body clearance. The influence of the input parameters on the output PK parameters AUC_0‐inf_ total body clearance for AT‐RvD1 in venous blood plasma and saliva in human simulation was performed at 0.1, 0.25, and 0.5 mg/kg doses. The sensitivity was determined as the mean of several sensitivities based on different relative variations, which were defined by multiplication of the value used for the simulation with variation factors (Kim et al., 2018).

## 3 RESULTS

The default mouse physiological parameters in PK‐Sim® 7.4 were modified to represent NOD/ShiLtJ mice. Specifically, the parameters weight, change in weight with relationship to age and gender, were altered based on Mouse Strain Datasheet‐001976 provided by the supplier, the Jackson Laboratory (Laboratory, 2018b). For developing a human PBPK model, the Caucasian American setting was selected from among the options prepopulated within the PK‐Sim® database. For both mouse and human species, anatomical and physiological parameters relevant for the model building included organ weights, blood flow rates, organ volumes, body mass index, hematocrit, and organ vascularization. A partial list of anatomical and physiological parameters of NOD/ShiLtJ mouse and humans with corresponding values are provided in Table 2. The human values in Table 2 represent an adult weighing 80.35 kg with a body mass index of 25.22 kg/m^2^.

**Table 2:** Anatomical and physiological parameters of NOD‐ShiLtJ mice and humans

Parameter	NOD/ShiLtJ mouse	Ref.	Human	Ref.
Average weight	27.5 g	(Laboratory, 2018a)	80.35 kg	(NHANES, 1997)
Body mass index (BMI)	N/A		25.22 kg/m^2^	(NHANES, 1997)
Hematocrit	0.45	(Davies & Morris, 1993)	0.45	(Davies & Morris, 1993)
Organ blood flow rate (L/min)		PK‐Sim® default		(Edginton, Schmitt, & Willmann, 2006)
Brain	0.00017		0.78	
Heart	0.000366		0.26	
Kidney	0.0017		1.32	
Liver	0.000458		0.42	
Lung	0.00711		6.25	
Spleen	0.00018		0.17	
Organ volumes (ml)		PK‐Sim® default		PK‐Sim® default
Brain	0.22		1,509.06	
Heart	0.12		416.52	
Kidney	0.45		437.33	
Liver	1.70		2,366.5	
Lung	0.13		1,210.12	
Spleen	0.13		206.38	
Saliva	0.00226		6.6	

Abbreviation: PK, pharmacokinetics.

We chose to perform initial simulations with intravenous route of administration due to the availability of formulation information of AT‐RvD1 as intravenous injection from previous studies (C. S. Wang et al., 2017). After the current intravenous model is validated and more information on alternative dosage forms of AT‐RvD1 is obtained, we will simulate AT‐RvD1 PK with alternative routes of administration.

**Figure 3:**
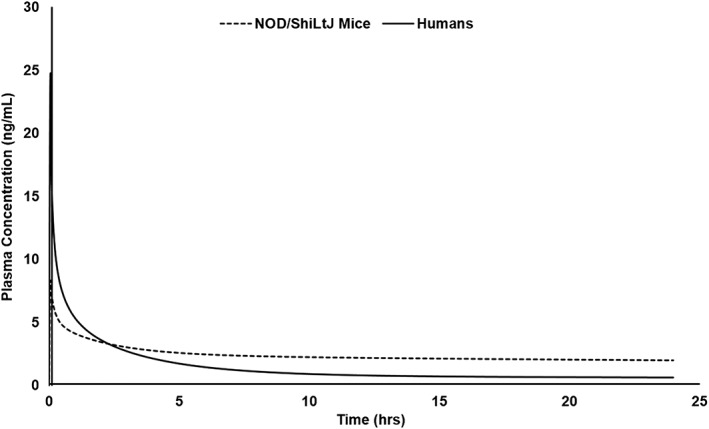
Simulated plasma concentration versus time curves of AT‐RvD1 in NOD/ShiLtJ mice and humans. AT‐RvD1 was administered intravenously at 0.1 mg/kg for the simulations (*n* = 100 in each species). AT‐RvD1, aspirin‐triggered resolvin D1

**Figure 4:**
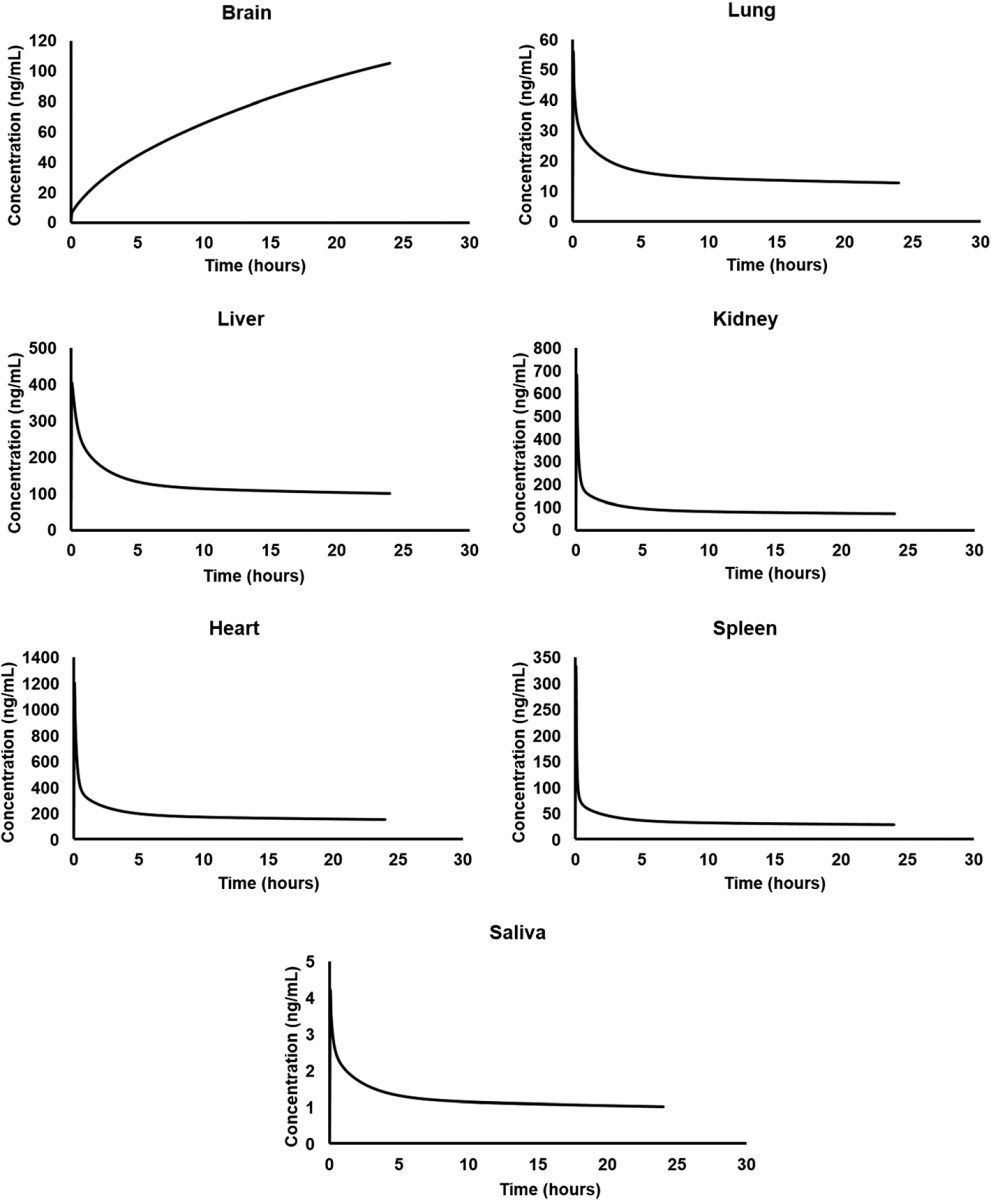
Simulated tissue concentration versus time profiles of AT‐RvD1 in NOD/ShiLtJ mice. AT‐RvD1 was administered intravenously at 0.1 mg/kg for the simulations (*n* = 100). AT‐RvD1, aspirin‐triggered resolvin D1

The predicted plasma concentration versus time curves of AT‐RvD1 after single intravenous administration at a 0.1 mg/kg dose in NOD/ShiLtJ mice and humans are provided in Figure 3. AT‐RvD1 was detected in all tissues included in the simulation as shown in Figure 4. The highest concentration of AT‐RvD1 was observed in the heart in comparison with the other organs. The PK analysis data for both NOD/ShiLtJ and humans are provided in Table 3.

**Table 3:** Simulated PK parameters of AT‐RvD1 in NOD‐ShiLtJ mice (*n* = 100) and humans (*n* = 100)

PK parameter	NOD/ShiLtJ mice	Human
*C*_max_ (ng/ml)	8.24 ± 0.14	24.26 ± 3.21
*T*_max_ (hr)	0.05	0.05
Half‐life (*t*_1/2_) (hr)	104.58 ± 0.27 0	91.13 ± 24.16
Elimination rate constant (Ke) (hr^1^)	0066 ± 0.00177	0.0076
[AUC]_0‐∞_ (ng*hr/ml)	363.13 ± 0.83	123.16 ± 35.1
MRT (hr)	148.01 ± 0.36	110.62 ± 33.67
Clearance (ml/min/kg)	4.58 ± 0.01	14.02 ± 2.97
Volume of distribution (Vd) (L/kg)	41.54 ± 0.01	118.91 ± 42.03

Abbreviations: AUC, area under the curve; MRT, mean residence time; PK, pharmacokinetics.

In NOD/ShiLtJ mice, the plasma concentration of AT‐RvD1 reached its maximum value (*T*_max_) by 3 min, with a half‐life of 104.58 hr noted. From the tissue distribution simulations, the highest *C*_max_ of AT‐RvD1 in NOD/ShiLtJ mice simulations was found in the heart tissue (1,180.43 ng/ml). This may be due to selecting AT‐RvD1 administration as for an intravenous route in the simulations. The simulated tissue distribution curves of AT‐RvD1 in NOD/ShiLtJ mice are provided as Figure 4.

**Figure 5:**
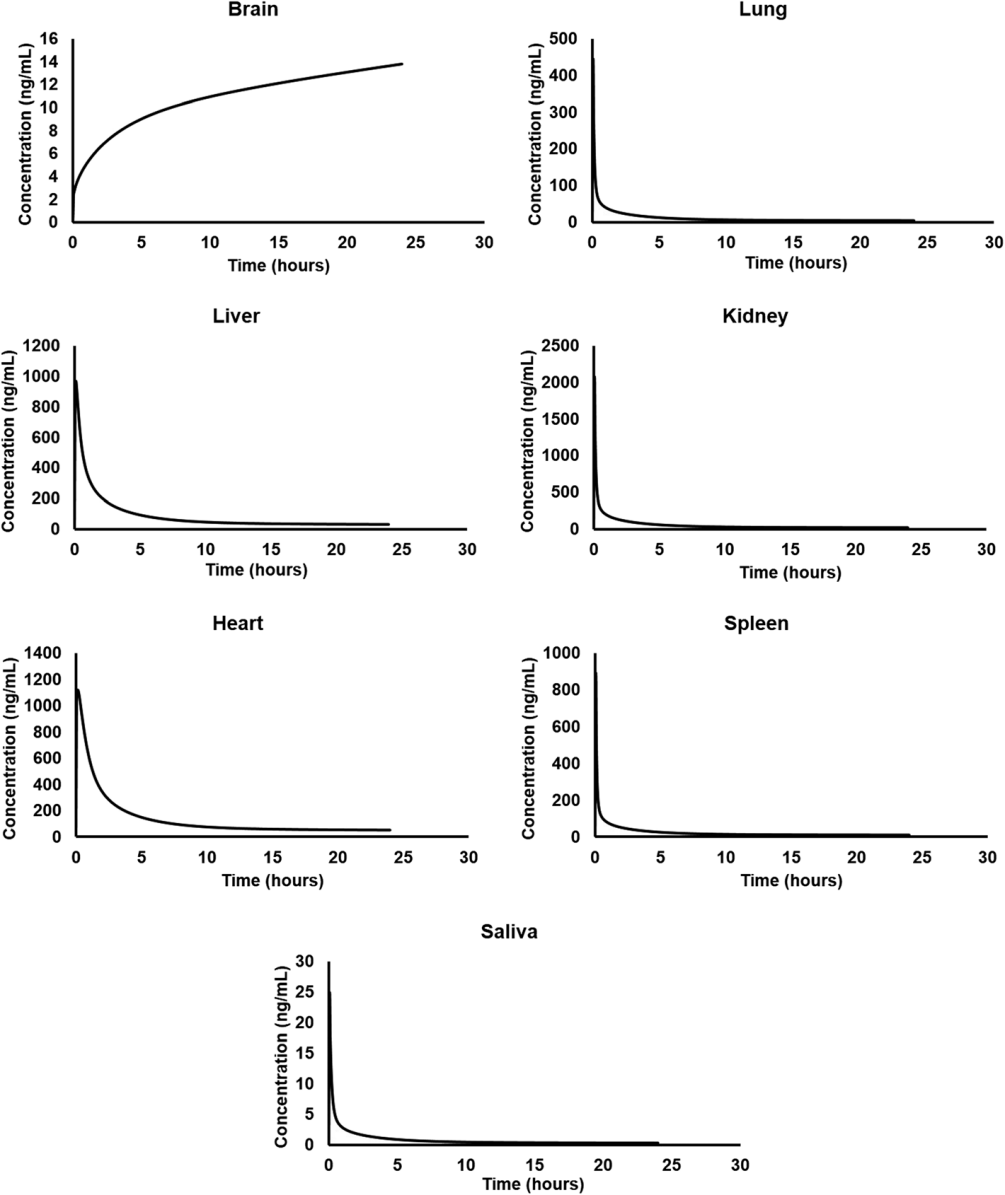
Simulated tissue concentration versus time profiles of AT‐RvD1 in humans. AT‐RvD1 was administered intravenously at 0.1 mg/kg for the simulations (*n* = 100). AT‐RvD1, aspirin‐triggered resolvin D1

The simulations of AT‐RvD1 in humans showed a *T*_max_ of 30 min and a half‐life of 91.13 hr. Interestingly, in human simulations, AT‐RvD1 showed to have the highest *C*_max_ in the kidneys at 2,036.06 ng/ml. AT‐RvD1 concentrations in saliva were also simulated in human simulations due to their relevance in SS. The simulations showed that AT‐RvD1 is present in saliva with a *C*_max_ of 24.28 ng/ml. This indicates that AT‐RvD1 reaches salivary glands at therapeutic concentrations, which is important for its development as a therapeutic agent for the treatment of SS. The simulated tissue distribution curves of AT‐RvD1 in humans are provided in Figure 5.

**Figure 6:**
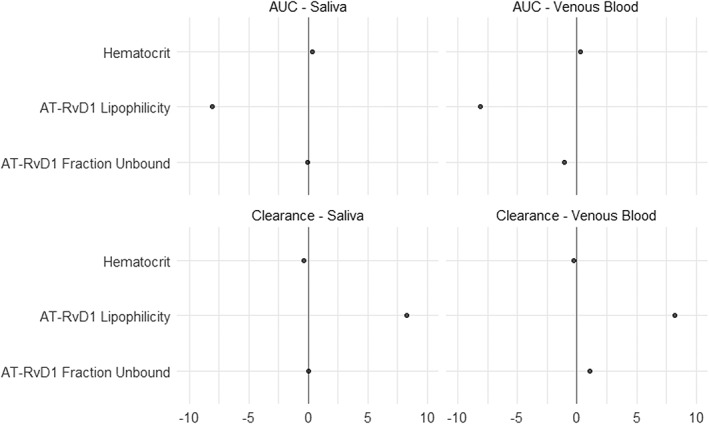
Sensitivity analysis of pharmacokinetics parameters AUC and clearance (plasma and saliva). Results are presented on model input parameters lipophilicity, hematocrit, and fraction unbound. AT‐RvD1, aspirin‐triggered resolvin D1; AUC, area under the curve

The results from the sensitivity analysis showed that both AUC_0‐inf_ and total body clearance of AT‐RvD1 in plasma and saliva were identified to be significantly sensitive to LogP across all doses that used for simulations. In addition, the AUC_0‐inf_ and total body clearance in plasma and saliva were also influenced by AT‐RvD1 fraction unbound in plasma and by hematocrit value, respectively. Figure 6 shows the results of sensitivity analysis performed at 0.1 mg/kg dose on output parameters AUC_0‐inf_ and total body clearance in plasma and saliva.

## 4 DISCUSSION

AT‐RvD1 has shown great potential for development as a therapeutic treatment of SS; however, mechanisms of these actions have yet to be determined. PK studies are a good option for determining these mechanisms, a method that is typically employed in the drug development process. However, such studies are expensive and time consuming; by contrast, computer modeling using PBPK approach offers the potential to virtually explore candidate pathways and offer predictions for which should be explored through subsequent confirmatory in vivo studies. Though the predictions made within this modeling study remain as yet untested within the laboratory, our findings nonetheless offer an enticing option for using an emerging technology to better target mechanistic studies, thereby increasing the explanatory power and efficiency of in vivo exploration. As such, dentists and dental researchers would benefit from knowledge of the potential offered by computer modeling for increased targeting of in vivo studies, with demonstration of such models in reference to AT‐RvD1 provided herein and discussed in detail below.

One of the advantages of using a PBPK modeling software such as PK‐Sim® is that the program incorporates default values for a mean representative of a species. These default values were carefully selected from the literature. In our study, the default anatomical and physiological values of mouse species that were provided in PK‐Sim® were modified to represent the values of NOD/ShiLtJ mice. Such species‐specific model parameters of NOD/ShiLtJ allow for better approximation of output values when compared with the default values. After model input parameters were finalized, the simulations were performed on virtual populations of NOD/ShiLtJ mice and humans. Virtual populations of 100 subjects were chosen for both NOD/ShiLtJ and human simulations to incorporate the influence of individual variability on the pharmacokinetics of AT‐RvD1 (Willmann et al., 2007). Furthermore, we utilized a dose of 0.1 mg/kg for both mice and human simulations in light of prior studies indicating that this dosage successfully reduced hyposalivation in NOD/ShiLtJ mice with SS‐like features. Sensitivity analysis results showed that both AUC_0‐inf_ and total body clearance were significantly influenced by the LogP of AT‐RvD1. Therefore, LogP needs to be adjusted using parameter identification to improve model predictions after obtaining observed data from a pharmacokinetic studies.

The data from simulations provided valuable information on PK of AT‐RvD1 in both mice and humans. In addition to plasma concentrations, we also obtained PK of AT‐RvD1 in highly perfused tissues such as the brain, heart, liver, lungs, spleen, and kidneys. As expected, AT‐RvD1 was well distributed in the simulated organs. Specifically, in the mouse model AT‐RvD1 accumulated in the heart and human model, it accumulated in the kidneys. The PK data showed a long half‐life for AT‐RvD1 in both mice and humans and the long plasma half‐life for AT‐RvD1 perhaps being attributable to absence of cytochrome P450‐based metabolism of AT‐RvD1 in the current model. Our findings are in accordance with biodistribution pattern of docosahexaenoic acid (DHA) in humans where DHA was reported to be accumulated at high concentrations in the kidney, liver, heart wall, spleen, and lungs (Umhau et al., 2009). Our biodistribution comparisons are only qualitative as quantitative comparison is not feasible due to differences in species and methods of quantification of DHA. The plasma levels of DHA were reported to be. The PK‐Sim® allows for simulating the PK of drugs in saliva in human species as a default compartment. However, for NOD/ShiLtJ mouse species, additional saliva compartment was added prior to simulations using MoBi® 7.4. The AT‐RvD1 simulations in mouse and human saliva are important as SS involves hyposalivation due to malfunctioning of salivary glands. The data in both NOD/ShiLtJ mouse and human simulations showed that AT‐RvD1 is present in saliva and followed a PK pattern similar to that of PK in plasma. The similarity between saliva and plasma PK of AT‐RvD1 is expected due to its lipophilicity (LogP = 3.22), with a well‐established body of literature indicating that lipophilic drugs such as caffeine, azithromycin, and valsartan showed salivary PK that is comparable with plasma PK (Dobson et al., 2016; Idkaidek, Agha, & Arafat, 2018; Idkaidek, Arafat, Hamadi, Hamadi, & Al‐Adham, 2017). As shown in Table 3, the PK parameters of AT‐RvD1 differ between mice and humans. Specifically, it is important to understand the mechanistic basis of these interspecies differences in AT‐RvD1 to ascertain the mechanism of disposition of AT‐RvD1. For example, the clearance of AT‐RvD1 in humans (14.02 ml/min/kg) is significantly higher when compared with the clearance in mouse (4.58 ml/min/kg). The mechanistic reasoning of this difference needs to be further investigated. However, one reason may be that the abundance values of the enzyme EOR in the mouse organs such as the liver and kidneys may not be accurately represented and therefore contributing to the underprediction of the clearance parameter.

The next step of this research involves validation of the mouse NOD/ShiLtJ PBPK model with in vivo PK data. Also, a detailed study of metabolic pattern of AT‐RvD1 with respect to major metabolic enzymes such as at cytochrome P450 must be performed to validate the model. Once the animal model is validated and established, the physiological parameters can be substituted to make the first prediction in humans. The data from the first prediction in humans can then be used to design first‐in‐human clinical trials to obtain human PK data. This human data can then be utilized to validate the human AT‐RvD1 PBPK model. Using this iterative “learn and confirm” approach as outlined in Figure 2, the model can be validated and utilized to predict PK of AT‐RvD1 at various doses, in different formulations, and by way of multiple routes of administrations. Therefore, the knowledge obtained from such simulations will aid in accelerating and streamlining the development process of AT‐RvD1 at both preclinical and clinical stages.

In summary, a PBPK model was developed to describe the PK of AT‐RvD1 in NOD/ShiLtJ mice and humans. Short‐term utility of pharmacokinetic modeling findings involve improved targeting of in vivo studies in that AT‐RvD1 follows a one‐compartment pharmacokinetic pattern after intravenous administration with a long elimination half‐life and rapidly distributes into highly perfused tissues such as the liver, lungs, kidney, brain, heart, and spleen, with highest concentrations in the heart, both of which are testable hypotheses that can and should be confirmed with subsequent studies. Likewise, the long‐term utility of these points as revealed by pharmacokinetic modeling is the potential for improved drug development and/or better designs for clinical trials.

## CONFLICT OF INTEREST

No conflicts to declare.
